# *In silico* analysis of expression data during the early priming stage of liver regeneration after partial hepatectomy in rat

**DOI:** 10.18632/oncotarget.24370

**Published:** 2018-01-27

**Authors:** Li Yin, Xueqiang Guo, Chunyan Zhang, Zhihui Cai, Cunshuan Xu

**Affiliations:** ^1^ College of Life Science, Henan Normal University, Xinxiang 453007, Henan Province, China; ^2^ State Key Laboratory Cultivation Base for Cell Differentiation Regulation and Henan Engineering Laboratory for Bioengineering and Drug Development, Henan Normal University, Xinxiang 453007, Henan Province, China; ^3^ Luohe Medical College, Luohe 462002, Henan Province, China

**Keywords:** liver regeneration, priming stage, in silico analysis, GSEA, Cnot3

## Abstract

The priming stage is the first step of liver regeneration (LR). This stage is characterized by the transition from G0 to cell cycle for 4 hours in rat. In this study, individual gene level and gene set level (GSEA) was performed to identify the candidate genes and significantly changed biological processes at 2 h after partial hepatectomy (PH). The leading edge analysis is performed to identify the key genes and iRegulon was employed for transcription factor (TF) analysis. A total of 53 differentially expressed genes were identified using RMA package based on R language at 2 h after PH, including the transcription factor, enzyme and cytokine. As the most important genes in our analysis, Socs3 was selected with a special analysis so as to find the pathways correlate to the expression of it. The changed significantly pathways in LR involved response to stress, ATP metabolism, and regulation of cell cycle mainly. Several transcription factors were identified including *Stat5a, Cnot3* and *zfp384*. Taken together, at the early priming stage of LR in rat, the liver is experiencing some changes including response to stress, activated ATP metabolism and inhibition of cell cycle. Our analysis provided a detailed and comprehensive map for further research of the early priming stage of LR in rat.

## INTRODUCTION

Liver plays roles in the metabolism of carbohydrates, lipid, proteins and biochemical defense against toxic chemicals. It is an amazing organ due to its high regenerative capacity in all vertebrate organisms. 2/3 partial hepatectomy (PHx) is a common surgical method in the study of liver regeneration. After the resection three of the five lobes, the remnant liver could grow to restore the mass of original lobes through proliferation of mature adult hepatocytes and the other hepatic cell types which is called liver regeneration (LR). The partial hepatectomy model is a powerful complementary tool for the identification of regulatory pathways involved in liver cell growth which are likely relevant to the pathogenesis of hepatocellular carcinoma [1].

The initiation of liver regeneration may be associated with several events including the increase in portal flow per unit liver, the activation of urokinase, the migration of catenin, the activation of notch and Wnt signaling. Many genes activate after PH which are not expressed in normal hepatocytes [2]. But all of the details are not clear. It is assured that each signal plays its meaningful role in different aspects in LR. Understanding this mechanism of the initiation of LR is very important. Previous studies have suggested that HGF,TNF,IL-6,STAT3,NFKB have impact on the process. The priming was defined as the transition of quiescent hepatocyte into the cell cycle by Fausto [3]. And the priming stage is species-dependent [4]. In this phase, the early period of 0-4 h after PHx, some events occurs preparing for the next progression [4]. In order to explore the changes and interaction mechanism of biological process , key genes and transcription factors (TFs) at the early priming stage in rat, especially at 2 h after PH, we done the gene set enrichment analysis mainly to find the intrinsic events which is important not only in experimental conditions but in clinical settings.

## RESULTS

### Data processing and DEGs identification

**Figure 1:**
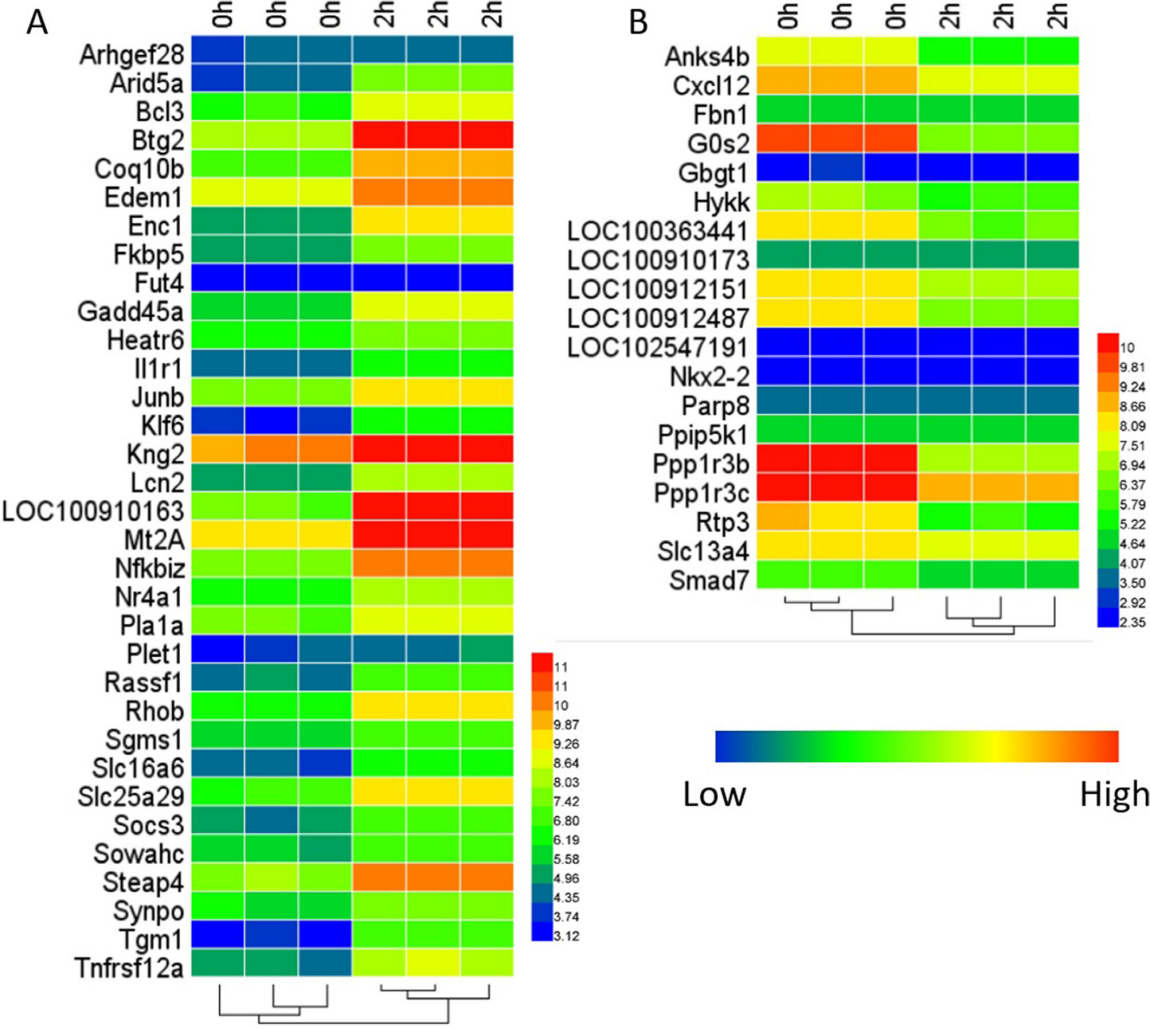
Heat-map of the DEGs (**A**) is for 33 up-regulated genes and (**B)** is for 19 down-regulated genes.

As a result, 31,099 probe sets mapped to 15,372 gene symbols under GPL 1355 platform. A total of 52 DEGs (Differentially expressed genes) were identified at 2 h after PH compared with the normal group, including 33 up-regulated genes and 19 down-regulated genes and the heat-map of DEGs is shown in Figure 1A and 1B. (Table 1).

**Table 1:** The classification of 53 DEGs in liver regeneration at 2 h after partial hepatectomy

Type	Symbol
cytokine	Cxcl12
enzyme	Edem1, Fkbp5, Fut4, Gbgt1, Pla1a, Rhob, Sgms1, Steap4, Tgm1
kinase	Hykk
ligand-dependent nuclear receptor	Nr4a1
peptidase	Enc1
phosphatase	Ppip5k1, Ppp1r3c, Socs3
transcription regulator	Anks4b, Arid5a, Bcl3, Btg2, Junb, Klf6, Nfkbiz, Nkx2-2, Smad7, Sowahc
transmembrane receptor	Il1r1, Tnfrsf12a
transporter	Lcn2, Slc13a4, Slc16a6, Slc25a29
other	Arhgef28, C8orf4, Coq10b, Fbn1, G0s2, Gadd45a, Heatr6, Loc100910173, Loc100912151, Mt2a, Parp8, Plet1, Ppp1r3b, Rassf1, Rtp3, Synpo

### Identification of biological pathways and process discriminating 2 h after PH from 0 h

**Figure 2:**
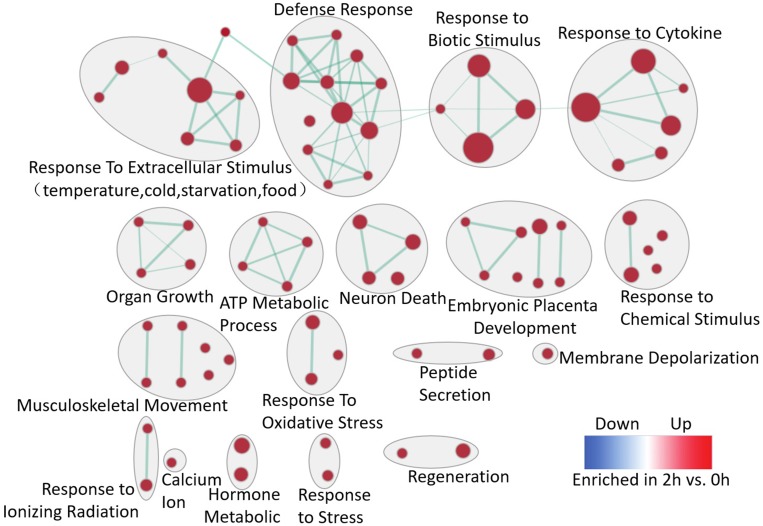
Enrichment map for the regenerative liver at 2 h The map displays the enriched gene-sets at 2 h vs. 0 h in LR. Nodes are colored according to enrichment results: red represents enrichment at 2 h, blue represents enrichment at 0 h; color intensity is proportional to enrichment significance. Clusters of functionally related gene-sets were manually circled and assigned a label. (FDR *q*-value < 0.1, *p*-value < 0.005).

The computational method, GSEA was used to identify over-represented biological pathways. The dataset has 31099 native features and there are 13343 genes after collapsing features into gene symbols. 2740 gene sets were used in the analysis after the size filtering (min = 15, max *=* 500). As a result, 85 gene sets enriched in 2 h after PH passing the threshold (*q*-value < 0.1, *p*-value < 0.005) were selected to perform enrichment visualization in Cytoscape as showed in Figure 2. The results showed that response to extracellular stimulus, biotic stimulus, cytokine, chemical stimulus, oxidative stress, ionizing radiation and stress, ATP metabolic process and organ growth is globally stronger at 2 h after PH. Remarkably, embryonic placenta development and regeneration are enriched at 2 h which obviously related to liver regeneration. It may be inferred that the primary stage of liver regeneration is similar to embryonic placenta development.

### Identification of key genes

**Figure 3:**
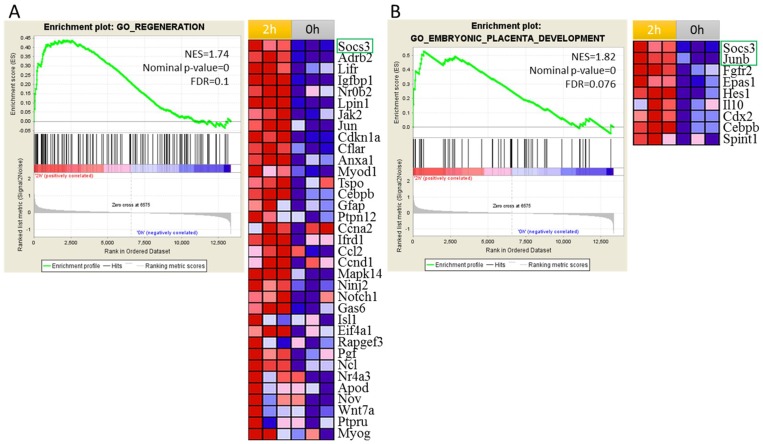
Gene set enrichment analysis (GSEA) identified regeneration and embryonic placenta development processes were activated at 2 h after PH In the plot, genes are ranked by signal/noise ratio according to their differential expression between 2 h after PH and 0 h. Genes in the gene set are marked with vertical bars. The normalized enrichment score(NES),nominal *p*-value and FDR are shown in the plot. (**A**) GSEA showed that the regeneration was up-regulated at 2 h after PH. Leading-edge analysis revealed the up-regulation of 35 genes that are important for regeneration according to their signal/noise ratio. (**B**) GSEA showed that embryonic placenta development was up-regulated at 2 h after PH. Leading-edge analysis revealed the up-regulation of 9 genes that are important for embryonic placenta development according to their signal/noise ratio. Genes in green box are DEGs.

**Figure 4:**
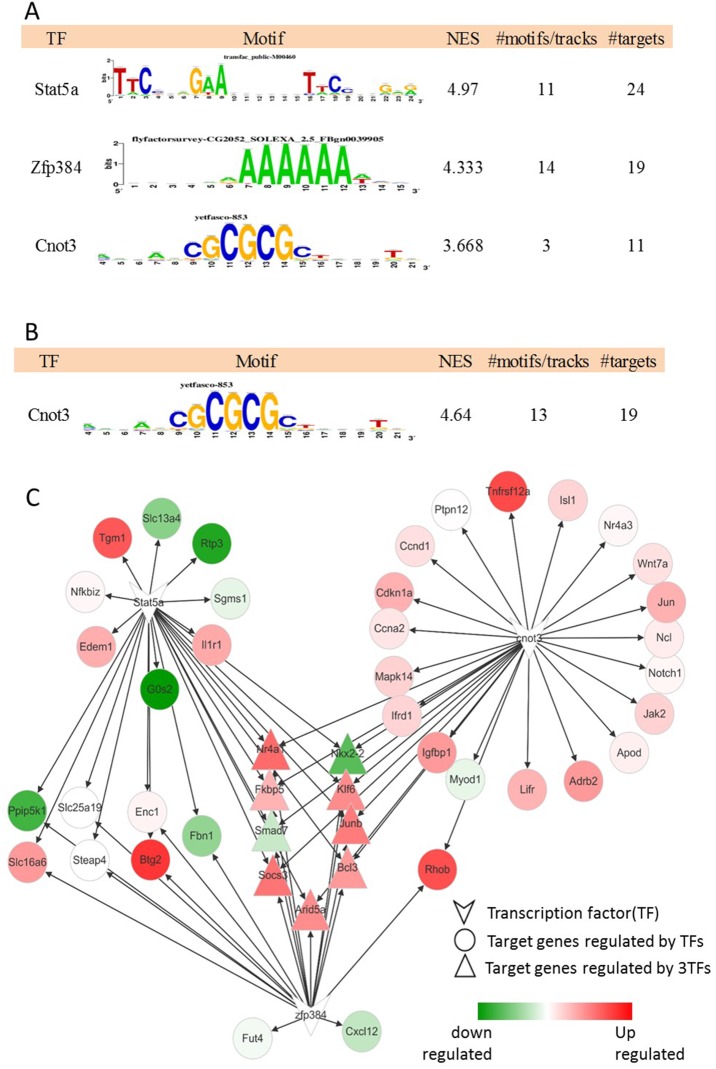
Master regulators at 2 h after PH and the gene regulatory network (**A**) The result of regulatory analysis with iRegulon on the DEGs and the regulatory network (**B**) The result of regulatory analysis with iRegulon on 35 genes contributed to the regeneration core enrichment and the regulatory network. (**C**) The regulatory network of 3TFs. The color is consistent with the logFC of each gene.

Because of the hierarchy nature of GO, different biological process could contain the same or similar genes. In order to demonstrate which genes appeared frequently in significantly enriched genes sets passing through the thresholds and explore the genes that have the highest impact on liver regeneration, 85 gene sets were selected to perform the leading edge analysis as shown in Figure 3 (Supplementary Table 1). Among the top 30 genes, only Socs3 and JunB belonged to DEGs. Then, we analyzed the regeneration and embryonic placenta development gene sets as showed in Figure 4. Both the genes set are up-regulated at 2 h after PH. As showed in the gene list which contributed the core enrichment, Socs3 appeared in two gene sets. All the above indicated that Socs3 play a vital role in the initial onset of liver regeneration. High expression of Socs3 is consistent with previous study which showed that Socs3 is greatly induced during the first 12 h after PH.

Previous work suggested that hepatocyte DNA replication and cell cycle progression were enhanced which lead to accelerating liver regeneration in Socs3 knockout mice (h-KO) mice after two-thirds partial hepatectomy. More and more study indicated that Socs3 is a tumor suppressor except for the suppressor of cytokine signaling.

### The transcription factor analysis

The genes co-expressed or participates in the same biological process or disease may be regulated by the same or similar transcription factors (TF). In our study, the gene-based tool, iRegulon, was used to search the user-defined space for motifs enriched around the transcription start site (TSS) of the genes that we are interested. These genes included DEGs and the ones which participated in the regeneration process. In 52 DGEs, the most enriched TF motifs were those for Stat5a, Zfp384 and Cnot3 with normalized enrichment score (NES) 4.97, 4.33, 3.66 respectively as showed in Figure 4A and Figure 4B. In the regeneration gene set, the most enriched motif was for Cnot3 with NES 4.64 and 19 target genes. The gene regulatory network was constructed based on the three TFs as shown in Figure 4C. The target genes regulated by Cnot3 were all up-regulated almost. And, 9 genes were co-regulated by three TFs with the triangle shown in the figure. Socs3 was regulated by three TFs.

### The enrichment analysis for the genes co-expressed with Socs3

**Figure 5:**
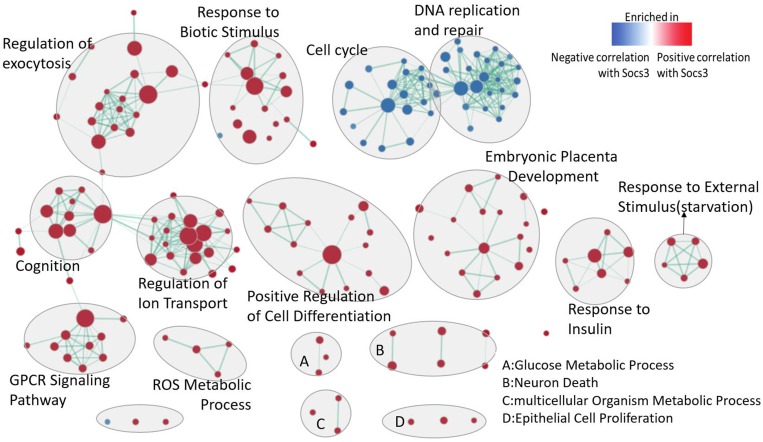
Enrichment map for the genes which correlate to the expression of SOCS3 at regenerative liver at all time-points The map displays the enriched gene-sets in positive and negative correlation with Socs3 respectively. Nodes are colored according to enrichment results: red represents enrichment in positive-correlated genes to Socs3, blue represents enrichment negative-correlated genes to Socs3. Clusters of functionally related gene-sets were manually circled and assigned a label. Pearson was selected for the Matirc for ranking genes. (FDR *q*-value < 0.1, *p*-value < 0.005).

The importance of Socs3 at 2 h after PH is obvious from the above analysis. So, we performed the gene set enrichment analysis for the Socs3 (1369584_at) phenotype. Briefly, in order to avoid the randomness of only one time-point we detected the gene sets correlated with Socs3 at all time-point after PH including 0,2,6,12,24,36,72 and168h (Supplementary Tables 2 and Supplementary Tables 3). The gene sets passing the threshold (*q*-value < 0.1, *p*-value < 0.005) were selected to perform enrichment visualization in Cytoscape as shown in Figure 5 (Supplementary Table 4 and Supplementary Figure 1). The genes which were positively correlated with the expression of Socs3 were enriched in response reaction, cell differentiation and embryonic placenta development mainly. The genes negatively correlated with the expression of Socs3 were enriched in cell cycle and DNA replication and repair.

## DISCUSSION

After removal of two-thirds of the liver, hepatocytes escape G0 phase and enter into G1 phase, DNA biosynthesis, mitosis and cytokinesis. The remnant liver will restore within two weeks. Some studies have suggested that the timing of hepatocyte entering into DNA synthesis after PH is species-dependent, which peaks 12-16 h earlier post-hepatectomy in the rat (20 h) compared with the mouse (32-36 h) [5]. And the reasons for that are not clear. Whatever rat or mice, the timing of the transition of the G0 hepatocyte into the cell cycle (priming stage) is 4 h [3]. The genome-wide expression profile of priming after PHx in mice has been documented. The authors detailed the differential expressed genes according to the different functions at the initial priming [4]. More and more researches suggested that IL6-Jak-STAT pathway was one of the most important events at the priming phase of liver regeneration.

BTG family member 2 (BTG2) is a p-53 inductive anti-proliferative protein. It induces the inhibition of cell cycle progression from G1 to S phase through Rb following DNA damage and other types of cellular stress [6–8]. Due to the overexpression of BTG2 at 2 h, it can be inferred that the signal for cell fate is defined at the early onset of LR or there may be other targets which BTG2 interact with at the priming stage.

As the member of the activating protein 1 (AP-1) transcription factor family, JunB differs from c-Jun in its DNA-binding and dimerization domains [9]. During early xenopus embryogenesis, JunB regulated the tail formation through FGF and Wnt signaling pathways [10]. In the study of relevance of JunB in multiple myeloma (MM), IL-6 may trigger the expression of JunB and the activation of MEK/ERK or NFKB is responsible for the induction of JunB expression and AP-1 transcriptional activity [11]. Knockdown of JunB (not c-Jun) in rat β-cells increased TNF-α+IFN-γ-induced apoptosis [12]. Though there are different even conflict results reported such as JunB can substitute for Jun in mouse development and cell proliferation in the absence of c-JUN, JunB is a negative regulator of gene expression [13]. Recent research showed that JunB plays a positive and non-redundant role in the induction of immune-related genes in macrophages [14]. Consistent with a previous study, JunB is induced at 2 h, which belonged to G0/G1 transition [15]. The mechanism of JunB is not clear.

Due to the deficiency of obvious DNA binding motif and the no obvious structural character has been detected among the genes which regulated by Nfkbiz, it may play its role more likely by stabilizing or assisting the promoter binding of other transcription regulators [16]. The over expression of Nfkbiz inhibit the transcriptional activity of Stat3 by the interaction between its C-terminal and the coiled-coil domain of Stat3, thus, Nfkbiz regulates proliferation and apoptosis by inhibiting the binding of STAT3 to DNA [16, 17]. The up-regulated Nfkbiz is DNA-damage induced and oncogene-induced in several models of senescence. The knockout of Nfkbiz impaired the expression of IL6 and IL8 [18]. In our study, the overexpressed Nfkbia may inhibit the activity of Stat so as to inhibit the cell proliferation at 2 h after PH.

The homeodomain transcription factor NK2 homeobox2 (Nkx2-2) is critical for cell fate decisions in pancreatic islet, enteroendocrine cell lineages, etc [19].

As the member of zinc finger transcription factor, the C terminal of Klf6 binds the GC rich DNA sequences. The overexpression of Klf6 may play a crucial role during nerve regeneration [20, 21].

Smad7 may block xenopus embryos development by blocking TGF-beta signaling, at the same time, Smad7 can inhibit phosphorylation of Smad2 and Smad3 mediated by TGF- beta which indicated that Smad7 may participate in a negative feedback loop to control TGF-beta response [22]. Gene delivery of Smad7 markedly improved the regeneration and functional recovery of quarter-size liver grafts by blocking the activation of Smad2/3 and nuclear translocation [23]. AT-rich interactive domain-containing protein 5a (Arid5a) could regulate naive CD4+ T cell fate by stabilizing Stat3 (not Stat1 and Stat5) mRNA [24, 25]. And Arid5a may promote inflammatory process by stabilizing IL-6 through binding the 3’ untranslatable region of IL-6 mRNA [26]. B-cell CLL/lymphoma 3 (Bcl3) may induce cell proliferation and inhibit cell death as an oncogenic gene by regulating STAT3 in several cancers including cervical cancer, glimo cell [27, 28]. At the same time, as the homology protein with IkappaB, Bcl3 may interact with Nfkb [29].

The Slc16a family of monocarboxylate transporters (MCTs) contains 14 members which related to monocarboxylate transport, homeostasis and fluid transport [30]. As the member of MCTs, Slc16a6 (MCT7) has not been functionally clear though the sequence homology to the known members [31] Slc25a29 is responsible for the importing of basic amino acids into mitochondria for the mitochondrial protein synthesis [32]. The solute linked carrier 13A4 gene (Slc13a4) expressed predominantly in human and mouse placenta with the function of nutrient sulfate to the fetus [33]. Because sulfate came from the metabolism of sulfur-containing amino acid could not satisfy the need of fetus, the loss of Slc13a4 could result in severe fetal abnormalities and death in mice in placenta [33, 34].

Lipocalin-2 (Lcn-2) expressed dramatically after PH. The normal number of hepatocytes from remaining one-third of the liver was responsible for 63% of the serum Lcn2 protein which is dependent on IL-6 activation [35–37]. Lcn2 plays a vital role in the restoring of liver after PH through a “help-me” signal [38]. Previous studies suggested that Lcn2 may be a biomarker for liver regeneration, and many works needs to be done so as to elucidate the molecular mechanism of Lcn2 in liver regeneration [39]. The G0/G1 switch gene2 (G0S2) has shown multifaceted functions including cell cycle, apoptosis, inflammation and metabolism [40]. According to the study of hematopoietic stem cells (HSCs), G0S2 prevent the HSCs from entering the cell cycle by cell-autonomous manner [41, 42]. G0S2 may exert its anti-apoptotic function through engaging in dialogue with Bcl-2 in mitochondria [43]. In lipid metabolism, G0S2 inhibits the function of Atgl which is required for the lipolysis [40]. In our study, the largely downregulated G0S2 may meet the demand of energy and substrates for membrane biogenesis which are necessary for the following cell cycle. Six-transmembrane epithelial antigen of prostate 4 (Steap4) involves multifunctional process such as glucose intake, energy metabolism and inflammatory response [44]. In our study, the overexpression of Steap4 improves the glucose intake and mitochondrial function.

As the most frequently occurring DEGs in several enrichment process, the suppressor of cytokine signaling 3 (Socs3) is worth further discussion. The expression level of Socs3 is opposite to the cell cycle, which is consistent with previous studies, that is to say, hepatocytes undergo the first round of DNA synthesis which peaks at 24 h in rat liver regeneration [45]. Socs3 plays its role both in expression of acute phase response and hepatocyte proliferation in LR and involves in hepatocarcinogenesis as a tumor suppressor [46, 47]. Socs3-KO mice showed several results including the accelerated liver regeneration, the enhanced proliferation ability of hepatocytes even lack of growth factors, Socs3 deficiency influence both cytokine activity and cell proliferation and Socs3-KO mice were apt to develop HCC [46]. The deficiency of Socs3 lead to embryonic lethality in mice [48]. Socs3 plays its role mainly in IL-6-JAK-STAT3 axis as an inhibitor [47, 49, 50]. In mouse liver, Stat5 impaired liver regeneration by inducing the expression of cell cycle inhibitors such as Cdkn2b and Socs3 and the induced Stat5 may increase the activity of Stat1 and Stat3 [51, 52] . Response to stress such as starvation or intracellular ATP level, Cnot3 could be induced to regulate the expression of death-related genes which determine cell fate [53]. Cnot3 is necessary for self-renewal of embryonic stem cells and the silencing of it led to replication arrest at the early stage of colorectal cancers [54]. Nuclear matrix protein 4 (Nmp4,Zfp384) is a relatively new transcription factor which regulates matrix related-proteins. It may involve in the regulation of ribosome biogenesis, the expression of Aquaporin 5 (Aqp5) and IL-1β promoter. The role of it in LR is not clear yet. Notably, as the most irreplaceable contributor for LR, HGF, its activator hepatocyte growth factor activator (HGFA) and its receptor MET only upregulated slightly at 2 h after PH [55–57]. According to the latest research published on cell reports contributed by Stanford University scientists, as an injury-regulated protein which activates Hgf, Hgf could induce the transition of stem cells into GAlert and speed recovery in cases where the injury is expected [58].

The genes related to preparative events directly or indirectly for the entry of hepatocytes into the cell cycle were described. The precise roles of many of early-expressed genes are not clear. However, the changes in the priming stage should be viewed as serving both the entry into the cell cycle or preparing for the entry. The result from GSEA showed the enhanced biological process at the priming stage of LR. Taken together, the stress response, ATP metabolism, regeneration, depolarization of membrane and response to cytokine activated obviously. The primary trigger for the priming of LR is not clear yet. Nontheless, it is sure that many growth factors, cytokines and signaling participate in the initiation process. Through the upstream regulator analysis from the DEGs, we can found that the cytokines (Tnf, Il1b, Il6, Il1a) and growth factors (Egf, Ngf, Fgf2) are all predicted active (Supplementary Table 5) with several of them are well-known early-expressed genes in LR. From the enrichment analysis of Socs3 co-expressed genes, we can conclude that the genes positive to the expression of Socs3 were mainly related to the response stress and regeneration and the negative ones to the cell cycle and DNA replication.

## MATERIALS AND METHODS

### Microarray data analysis and identification of differentially expressed genes (DEGs) using limma package

The mRNA expression profile at 2 h after PH came from what we have done before and microarray data have been deposited in the GEO with the accession no.GSE55434. The platform is GPL1355 and all samples are hybridized on the Affymetrix Rat Genome 230 2.0 Array. The raw .CEL files were converted to expression data using Robust Muliti-arry Average algorithm (RMA) using the Bioconductor affy package. The quality control was performed using simpleaffy package. The DEGs are identified using a *t*-test in the limma package and the threshold for the DEGs was set adj-*p*value < 0.05 and |logFC| > 1.

### Gene set enrichment analysis (GSEA) and leading edge analysis

GSEA is a computational method that determines if an priori defined set of genes indicates statistically significant between two phenotypes [4, 2, 60, 59]. In GSEA, genes are ranked by their correlation with phenotype and every enrichment gene set will get an enrichment score (ES). In this study, we adopted two kinds of methods to perform GSEA. The first, the expression dataset is 2 h and 0 h and the phenotype labels is 2 h vs. 0 h. The second, we employed the time series data from 0 h to 168 h after PH including 0, 2, 6, 12, 24, 36, 72 and 168 h (all the raw file can be obtained in GEO) and the phenotype is Socs3 which is my favorite gene. 1000 gene permutations were used to generate a null distribution for ES, then each pathway will attain a normalization enrichment score (NES). C5:BP:GO biological process including 4436 gene sets are used as gene sets database. Gene sets with considered significantly enriched with *q*-value < 0.1 and *p*-value < 0.005.

### Enrichment map visualization and leading edge analysis

The functionally coherent gene-sets enriched by GESA may be statistically over-represented in a given gene list which may led to the gene-set redundancy. In order to interpret the output from GSEA, the enrichment map visualization was employed to integrate the result. The enrichment map was generated using only the gene-sets passing the thresholds: nominal *p*-value < 0.005, q-value < 0.1.The overlap coefficient was set to 0.5. A leading edge analysis was performed to elucidate key genes associated with the process what we are interested in.

### The construction of gene regulatory networks

Gene regulatory networks consist of interactions between TFs and their target genes. Every TF binds the specific DNA site near its target gene. The iRegulon plug-in was used to identify the master transcription factors (TFs) regulating DEGs and the biological processes based on binding motifs and track collections. Only the TFs scored highly were further studied. The gene regulatory network is built using Cytoscape 3.4.0 (http://cytoscape.org/).

## CONCLUSIONS

We analyzed the transcriptional expression profile at 2 h after PH in rat using silico tools in which the hepatocytes are undergoing G0-G1 transition. Different kinds of DEGs were identified including cytokine, enzyme, transcription regulator etc.. These genes have broad functions mainly in cell cycle inhibition, stress response, metabolism and membrane-related. Two kinds of GSEA were done to analyze the biological process at 2 h. One is globle-based and the other special phenotype-based (Socs3). The results showed that (a) stress response activated at 2 h after PH (b) cell cycle was inhibited (c) metabolism activated which is consistent with previous studies [4].

The activation of metabolism may be prepared for the requirement of substance and energy for following cell cycle. It can be inferred that liver regeneration is similar with embryonic development in some degree at 2 h which is not consistent with a previous study in some degree [59]. The changes at 2 h after PH in rat suggested that some cellular process and genes changed before DNA synthesis. Especially, some genes had expressed which regulate the following cell cycle. Namely, the suppressor of cell cycle transcription at the early priming stage of LR. The TF analysis based on the DEGs and genes from regeneration gene set was done and two relatively new regulators were identified including Cnot3 and Zfp384. Taken together, at the early priming stage of LR in rat, the hepatocytes are experiencing some changes including the response to stress, activated metabolism and inhibition of cell cycle. What is the primary –causes genes and process at priming stage of LR? What are the difference and similarity between LR and embryonic development at the priming stage? Future experiments may shed light on the liver diseases, regeneration and wound healing.

## SUPPLEMENTARY MATERIALS FIGURES AND TABLES










