# Supplementary material for: In silico analysis of expression data during the early priming stage of liver regeneration after partial hepatectomy in rat
Source: Oncotarget. 2018 Jan 27;9(14):11794–804. doi: 10.18632/oncotarget.24370 (PMC5837750; doi:10.18632/oncotarget.24370)
Supplement: Supplementary file 1 [file oncotarget-09-11794-s001.pdf]

In silico analysis of expression data during the early priming stage of liver regeneration after partial hepatectomy in rat

SUPPLEMENTARY MATERIALS

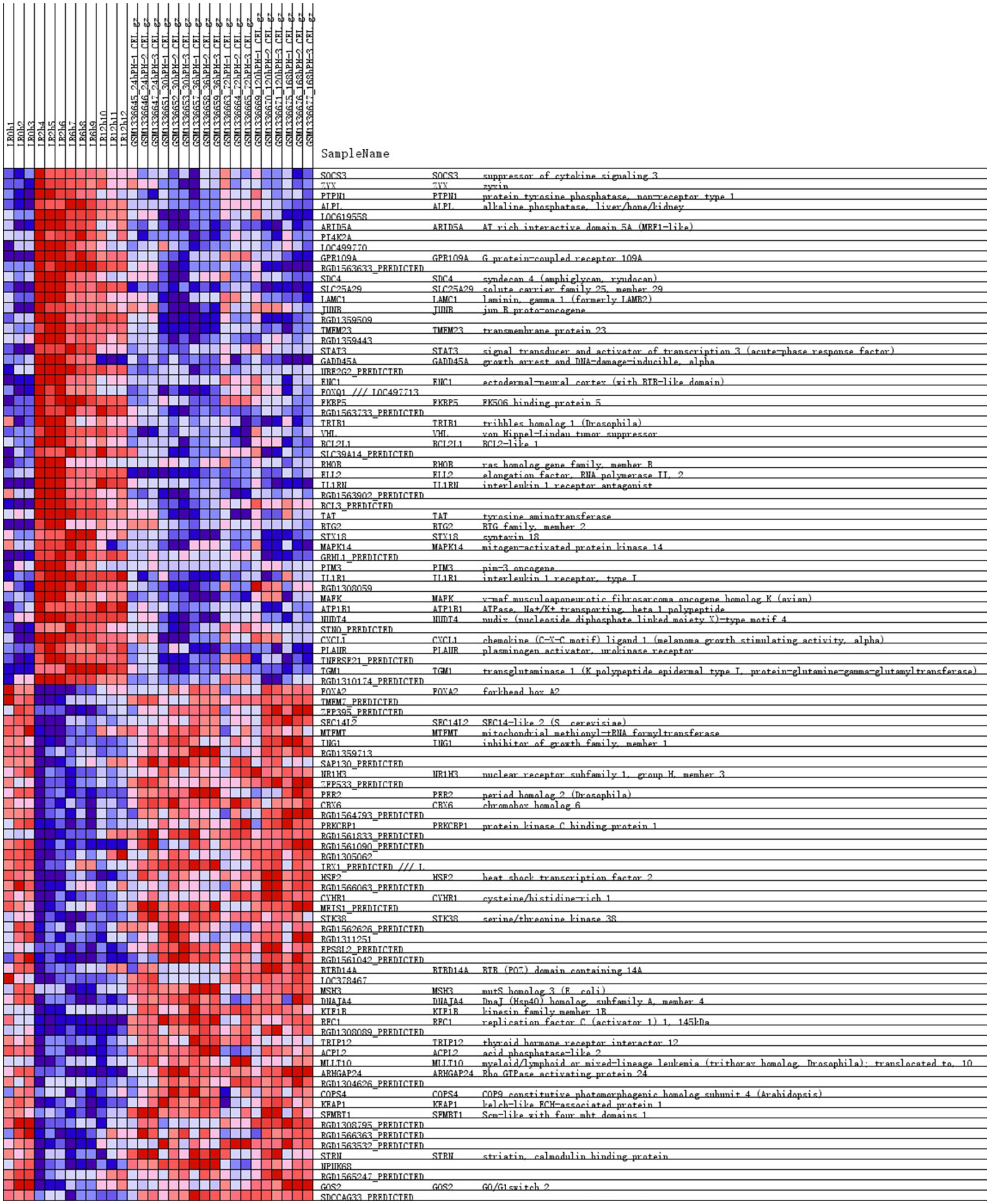

Supplementary Figure 1: The genes co-expressed with *Socs3* at all time-point after PH.

Supplementary Table 1: The leading edge analysis results for 83 genes. See_Supplementary Table 1

Supplementary Table 2: The gene sets correlated with Socs3 at all time-point after PH. See_ Supplementary_Table 2

Supplementary Table 3: The time-series phenotype file

**#numeric**
#0h
0 0 0
#2h
2 2 2
#6h
6 6 6
#12h
12 12 12
#24h
24 24 24
#30h
30 30 30
#36h
36 36 36
#72h
72 72 72
#120h
120 120 120
#168h
168 168 168

Supplementary Table 4: Ranked gene list 1369584_at positive versus 1369584_at negative. See_ Supplementary_Table 4

Supplementary Table 5: The results of upstream regulator analysis from DGEs based on IPA. See_Supplementary_Table 5
